# Genomic Insights Into Host Shifts Between *Plasmodium vivax* and *Plasmodium simium* in Latin America

**DOI:** 10.1111/eva.70222

**Published:** 2026-03-20

**Authors:** Margaux J. M. Lefebvre, Fanny Degrugillier, Céline Arnathau, Camila González, Silvia Rondón, Andrés Link, Andrea Chaves, Julio A. Benavides, Aline Alves Scarpellini Campos, Edmilson dos Santos, Rosana Huff, Cláudia Maria Dornelles da Silva, Ezequiel Vanderhoeven, Benoit De Thoisy, Michael C. Fontaine, Franck Prugnolle, Virginie Rougeron

**Affiliations:** ^1^ Department of Archaeogenetics Max Planck Institute for Evolutionary Anthropology Leipzig Germany; ^2^ MiVEGEC, Univ. Montpellier, CNRS, IRD Montpellier France; ^3^ Centro de Investigaciones en Microbiología y Parasitología Tropical (CIMPAT), Departamento de Ciencias Biológicas Universidad de los Andes Bogotá D.C. Colombia; ^4^ Laboratorio de Ecología de Bosques Tropicales y Primatología, Departamento de Ciencias Biológicas, Facultad de Ciencias Universidad de Los Andes Bogotá D.C. Colombia; ^5^ Centro Nacional de Innovaciones Biotecnológicas (CENIBiot), CeNAT‐CONARE San José Costa Rica; ^6^ Escuela de Biología Universidad de Costa Rica San Jose Costa Rica; ^7^ One Health Institute and Doctorado en Medicina de la Conservación, Faculty of Life Sciences Universidad Andrés Bello Santiago Chile; ^8^ Centro Estadual de Vigilância Em Saúde, Secretaria de Saúde Do Rio Grande Do Sul Porto Alegre Brazil; ^9^ Asociación Civil Centro de Investigaciones del Bosque Atlántico Puerto Iguazú Misiones Argentina; ^10^ Instituto de Biología Subtropical (IBS) Universidad Nacional de Misiones, CONICET Puerto Iguazú Misiones Argentina; ^11^ Institut Pasteur de la Guyane, Laboratoire Des Interactions Virus Hôtes Cayenne Guyane France; ^12^ Groningen Institute for Evolutionary Life Sciences (GELIFES) University of Groningen Groningen the Netherlands; ^13^ REHABS, International Research Laboratory, CNRS‐NMU, George Campus, Nelson Mandela University George South Africa; ^14^ Sustainability Research Unit, George Campus Nelson Mandela University George South Africa

**Keywords:** host jump, *plasmodium simium*, *plasmodium vivax*, population genomics, zoonotic malaria

## Abstract

Malaria in Latin America is largely caused by *Plasmodium vivax*, but the closely related monkey parasite *Plasmodium simium* has recently been observed in humans, thus raising new public health concerns. By screening 719 monkey samples from five Latin America countries, we identified 23 *Plasmodium*‐positives. However, only four samples yielded sufficient mitochondrial DNA sequencing data to allow reliable species identification, and their inclusion in genome‐wide population analyses. Using whole‐genome variation data from these samples together with whole genome variations of 19 *P. simium* and 405 *P. vivax* isolates, we investigated their evolutionary history and population genetics. *P. vivax* , typically restricted to humans, was identified in three Colombian and one Brazilian monkeys, suggesting possible host niche expansion. Genetic analyses reveal recent genetic exchanges between both species and indicate that *P. simium* originated from a host jump approximately one to two centuries ago. Also other alternatives are possible, this host shift may have followed *P. vivax* migration from Central/North America to Brazil. Genome‐wide scans revealed signals of positive selection in *P. simium* genes implicated in interactions with primate hosts, including *PvRBP2a* and *PvRBP1b*, as well as genes involved in interactions with mosquito vectors, such as *PvCMRP1*, *PvPAT*, and *Pvs47*. These findings shed light on *P. simium* evolutionary history. They also underscore the zoonotic risks, and the need to include monkeys in malaria prevention measures while ensuring human‐wildlife coexistence.

## 1 Introduction

*Plasmodium* species are responsible for malaria in a wide range of vertebrate hosts, including primates, rodents, reptiles, and birds (Garnham 1980). Within this genus, *Plasmodium falciparum* and *Plasmodium vivax* are the most clinically significant species in terms of malaria‐related morbidity and mortality in humans (World Health Organization, Geneva 2023). Other species, such as *Plasmodium malariae*, *Plasmodium ovale curtisi*, *Plasmodium ovale wallikeri*, *Plasmodium knowlesi*, *Plasmodium cynomolgi*, and *Plasmodium simium*, can also infect humans, contributing to the complexity of malaria epidemiology (Rougeron et al. 2021). *P. knowlesi* , *P. cynomolgi*, and *P. simium* primarily infect non‐human primates (NHPs), contributing to emerging zoonotic malaria cases (Baird 2009; Brasil et al. 2017; Su and Wu 2021). These are illustrations of “host jumps”, defined as the process by which pathogens settle and transmit in a host from a different species (Pedersen and Davies 2009). Today, zoonotic malaria, caused by malaria parasites transmitted from animals to humans, is becoming problematic due to human encroachment on forest habitats (Bueno et al. 2013; van de Straat et al. 2022). The increased risk of transmission from NHP reservoirs (Bueno et al. 2013; van de Straat et al. 2022) potentially challenges malaria elimination programs and makes zoonotic malaria a potential public health issue. The term zoonosis then refers to parasite transmission from non‐human primates to humans, and reverse zoonosis to describe transmission from humans to non‐human primates (de Oliveira, Rodrigues, Duarte, et al. 2021).

In South America, *P. simium* primarily infects platyrrhine monkeys (Rougeron et al. 2022). It was initially described in 1951 from blood smears of a southern brown howler monkey (*Alouatta clamitans*) near São Paulo, Brazil (Da Fonseca 1951), and since then it has been detected in other monkey species (de Oliveira, Rodrigues, Early, et al. 2021; Rougeron et al. 2021). Its zoonotic potential was first described in 1966 when human infections were identified in Brazil through blood smear analysis, raising concerns about cross‐species transmission (Deane et al. 1966). Historically, *P. simium* is believed to be endemic in Brazil (de Castro Duarte et al. 2008; Rougeron et al. 2022), and has more recently been implicated in human malaria cases (Brasil et al. 2017). Nevertheless, the detection of infections in NHPs from Colombia in 2019 (Rondón et al. 2019) and Costa Rica in 2022 (Chaves et al. 2022) suggested a broader distribution, although it remains unclear whether these infections were caused by *P. simium* or *P. vivax* .

Genetically, *P. simium* is closely related to *P. vivax* , although they can be distinguished by two mitochondrial single nucleotide polymorphisms (SNPs) and morphological differences in erythrocytic stages (Brasil et al. 2017; Ibrahim et al. 2023). In addition, no hybrid has been identified to date (Mourier et al. 2021), supporting the classification of *P. simium* as a distinct species from *P. vivax* (Da Fonseca 1951). However, due to their genetic similarities, shared host range (humans), and potential for recombination, the distinction between *P. simium* and *P. vivax* may not be as clear‐cut as currently understood and further investigations are required to clarify the taxonomic status of the two species. This ambiguity may reflect an incomplete or recent divergence. In this context, divergence refers to the evolutionary differentiation between lineages resulting from genetic change caused by differentiation of ecological niche, such as host jumps.

*Plasmodium simium* evolutionary origin(s) and history were largely overlooked until 2017 (Rougeron et al. 2021), when Brasil et al. (2017) characterized its mitochondrial genome following a human malaria epidemic in Brazil. They showed that this epidemic was caused by *P. simium* isolates. Then whole genome sequencing (WGS) studies confirmed that *P. simium* is genetically close to the American human *P. vivax* and characterized by a lower genetic diversity (de Oliveira, Rodrigues, Duarte, et al. 2021; Mourier et al. 2021), suggesting a host jump from human to Neotropical NHPs (de Oliveira, Rodrigues, Duarte, et al. 2021; de Oliveira, Rodrigues, Early, et al. 2021; Mourier et al. 2021) (i.e., reverse zoonosis (de Oliveira, Rodrigues, Duarte, et al. 2021)). Intriguingly, some studies showed that Brazilian *P. simium* strains are more closely related to Mexican than to Brazilian *P. vivax* , without providing any explanation for such results (de Oliveira, Rodrigues, Duarte, et al. 2021; Mourier et al. 2021). Other studies, based on mitochondrial genomes and some nuclear markers, suggested the occurrence of multiple host jumps (Lim et al. 2005; Tazi and Ayala 2011), that involved not only human *P. vivax* populations from Latin America, but also potentially from Asia (Carter 2003; Li et al. 2001). Additionally, although the genetic proximity of these species suggests a recent host jump (de Oliveira, Rodrigues, Duarte, et al. 2021; de Oliveira, Rodrigues, Early, et al. 2021; Mourier et al. 2021; Rougeron et al. 2022), to our knowledge, no precise date for this event has been provided. *P. simium* evolutionary history and origin(s) remain still unresolved.

The mechanisms by which *P. simium* has adapted to different hosts and vectors are also poorly understood. No study has explored the population genomics of the host jump from humans to NHPs. This is likely to have exerted strong selective pressures on the parasite, driving its adaptation to new hosts and resulting in distinct phenotypes and genotypes. Only some studies targeting candidate genes identified some genes that may have facilitated *P. simium's* successful invasion of the Neotropical primate hosts (de Oliveira, Rodrigues, Early, et al. 2021; Mourier et al. 2021). For instance, deletions were found in the coding regions of *pvrbpa2* (reticulocyte binding protein 2a) and *pvdbp1* (Duffy binding protein 1), two genes involved in the invasion of reticulocytes in primates (de Oliveira, Rodrigues, Duarte, et al. 2021; Mourier et al. 2021). Other than adapting to primate hosts, the parasite also has to adapt to the vector responsible for its transmission. In Latin America, *P. vivax* is mainly transmitted by *Anopheles darlingi* (Laporta et al. 2015; Zimmerman 1992) and *Anopheles albimanus* (Frederick et al. 2016; Zimmerman 1992). Conversely, it is thought that *P. simium* is transmitted by anopheline mosquitoes of the *Kerteszia* subgenus, primarily *Anopheles cruzii* and *Anopheles bellator* (Marrelli et al. 2007; de Pina‐Costa et al. 2014), suggesting adaptations to these distinct mosquito vectors. For example, *pvs47*, a gene involved in the evasion of the mosquito immune system, harbors two amino acid changes in *P. simium* that are absent in global *P. vivax* populations (de Oliveira, Rodrigues, Duarte, et al. 2021). However, a comprehensive genomic understanding of these adaptations is still lacking.

**FIGURE 1:**
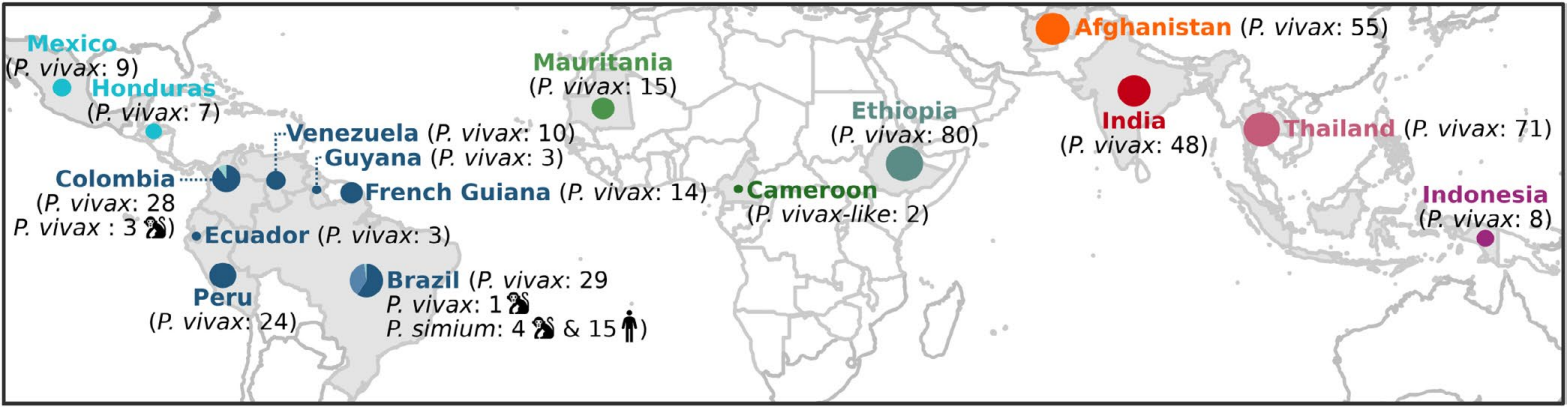
Geographic origins of the 408 *P. vivax* isolates, 19 *P. simium* isolates, and two African great apes *P. vivax‐like* isolates. The number of samples for each species is indicated between brackets and by the circle size. The monkey pictogram indicates *P. vivax* and *P. simium* isolates from non‐human primates in Latin America. For *P. simium* samples, the human pictogram indicates human samples.

Given the zoonotic potential of *P. simium*, understanding its geographic range, evolutionary origin(s), and adaptation mechanisms is essential for effective malaria control and elimination, but also for the conservation of endangered NHPs that might be affected by this pathogen. This study aimed to clarify *P. simium* geographic distribution and host range in Latin America and to investigate its evolutionary history and adaptations. To address these questions, we first screened 719 NHP samples from five Latin American countries (Colombia, Brazil, Argentina, Costa Rica, and French Guiana) for the presence of *P. simium*. This extensive screening identified three Colombian NHPs infected with *P. vivax* and not *P. simium*, a finding that has not been previously reported. We then analyzed whole genome variations for these samples and previously published *P. simium* (*n* = 19) and *P. vivax* (*n* = 405) datasets to assess population genetic diversity, structure, evolution history, and evidence of selection (Table S1 and Figure 1). To our knowledge, this represents the first comprehensive joint analysis of all available *P. simium* genomes. Unlike previous studies that excluded low‐coverage data (de Oliveira, Rodrigues, Duarte, et al. 2021), we incorporated these samples using methods that explicitly account for genotype uncertainty (Lou et al. 2021), thereby enabling the recovery of previously overlooked genomic variation. We found that the simian *P. vivax* samples from Colombia were more closely related to the Asian human *P. vivax* , whereas a Brazilian simian sample showed hybrid ancestry between *P. simium* and *P. vivax* . These results suggest the existence of an ongoing reverse zoonosis (i.e., *P. vivax* transfer from humans to Neotropical NHPs), which may in turn increase the risk of subsequent zoonotic malaria outbreaks. Furthermore, this is the first study to investigate and estimate the timing of the host jump leading to *P. simium*. Our population genetic inferences suggest that *P. simium* likely originated from a transfer of human *P. vivax* to Brazilian NHPs ~100–200 years ago. This may coincide with the migration of human *P. vivax* from Mexico to this region, although we can not rule out alternative hypotheses based on the current sampling. Lastly, we detected signals of positive selection in genes involved in interactions with primate hosts and mosquito vectors. Our results underline the importance of considering NHP as potential reservoirs of human malaria. This is crucial to anticipate potential emerging outbreaks and more globally to address human malaria public health issues but also the conservation of NHPs, who might be threatened by the disease transmission.

## 2 Results

### 2.1 Evidence of *P. vivax* Infections in South American Non‐Human Primates

Among 719 samples collected from NHPs across five Latin American countries (14 Colombian, 23 Brazilian, 30 Argentinian, 280 French Guianese, and 372 Costa Rican), 23 (3 from Colombia, 14 from French Guiana, and 6 from Brazil) were positive for *Plasmodium* using the *Cytochrome‐b* based PCR assay (Prugnolle et al. 2010) (Table S2). These 23 samples were derived from various biological materials (stool, liver, mixed liver and kidney, and blood) and collected from five NHP species ( *Alouatta macconnelli* , *Saguinus midas* , *Ateles hybridus* , *Alouatta clamitans*, and *Cebus versicolor* ) (see Table S1). All Colombian samples had previously been confirmed as PCR‐positive for *P. vivax* or *P. simium* in Rondón et al. (2019). For the remaining samples, the sequence analysis of the *Cytochrome‐b* gene revealed that they were all infected with either *P. vivax* or *P. simium*. All 23 *Plasmodium*‐positive samples were then sent for whole genome sequencing (WGS), after selective whole‐genome amplification (Cowell et al. 2017). Among these 23 samples, only four (three from feces and one from blood) provided sufficient mitochondrial DNA (mtDNA) sequencing depth to reliably distinguish between *P. simium* and *P. vivax* (see Material and Methods). These four samples were therefore integrated with previously published WGS data from 405 *P. vivax* and 29 *P. simium* genomes from previous studies to create a comprehensive dataset (Table S1, Figure S1). Of the 33 *P. simium* or undetermined ( *P. vivax* or *P. simium*) samples, 3 from Colombia (Col_164, Col_68 and Col_86 newly sequenced in the present study) and one from Brazil (P160 from de Oliveira, Rodrigues, Duarte, et al. (2021)) displayed mtDNA SNPs specific to *P. vivax* (Brasil et al. 2017; de Alvarenga et al. 2018; Ibrahim et al. 2023), thus indicating that *P. vivax* circulates among South American NHPs. However, interpretations regarding the evolutionary history of these simian isolates should be considered with caution given the limited number of samples (*n* = 4) and the heterogeneous sequencing depth among them (Table 1).

**TABLE 1:** Summary of sequencing coverage and single nucleotide variant sites included for *P. vivax* samples from non‐human primates and *P. simium* samples analyzed in this study.

Sample	Species	Host	Country	% of genome ≥ 1×	% of genome ≥ 5×	% of genome ≥ 10×	% of genome ≥ 30×	Median coverage (×)	Mean coverage (×)	Number of sites in the *dataset_GL*	Number of sites in the *dataset_HF*
143	*P. simium*	*H. sapiens*	Brazil	70.5	47.2	36.1	19.6	4	32.6	805,373	376,932
761	*P. simium*	*H. sapiens*	Brazil	27.3	15.7	13.0	9.3	0	166.5	277,549	82,850
2302	*P. simium*	*A. guariba*	Brazil	94.0	92.4	89.6	64.7	36	34.5	1,101,940	809,449
1272MT	*P. simium*	*H. sapiens*	Brazil	80.5	63.6	54.6	39.5	13	213.9	931,599	409,618
95_isolate	*P. simium*	*A. clamitans*	Brazil	60.6	10.6	1.8	0.1	1	2.5	675,603	67,372
97Ps	*P. simium*	*A. clamitans*	Brazil	40.9	23.2	17.6	10.8	0	34.6	442,051	157,554
AF1	*P. simium*	*H. sapiens*	Brazil	91.9	65.4	25.2	0.4	6	7.1	1,041,419	510,284
AF28	*P. simium*	*H. sapiens*	Brazil	93.0	86.4	74.6	24.3	18	21.2	1,080,694	685,791
AF30	*P. simium*	*H. sapiens*	Brazil	81.9	37.2	10.8	0.3	3	4.4	906,428	156,683
AF33	*P. simium*	*H. sapiens*	Brazil	94.9	88.8	69.3	2.1	13	13.6	1,097,243	758,474
Col_164	*P. vivax*	*A. seniculus*	Colombia	8.9	3.6	1.9	0.4	0	3.6	15,615	NA
Col_68	*P. vivax*	*A. hybridus*	Colombia	11.5	3.9	1.8	0.3	0	2.5	23,999	NA
Col_86	*P. vivax*	*A. hybridus*	Colombia	4.2	2.0	1.3	0.6	0	22.6	5641	NA
D121Ps	*P. simium*	*A. clamitans*	Brazil	38.7	19.8	13.8	7.1	0	13.5	417,044	148,963
MASM	*P. simium*	*H. sapiens*	Brazil	63.1	41.0	31.4	18.0	2	35.6	701,226	313,534
P160^a^	*P. vivax*	*C. nigrifrons*	Brazil	45.7	6.2	2.4	0.7	0	3.8	476,282	38,682
PVB_01._13	*P. simium*	*H. sapiens*	Brazil	69.5	32.0	15.8	3.0	2	6.0	840,917	267,739
PVB_01.19	*P. simium*	*H. sapiens*	Brazil	85.1	67.7	49.0	15.8	9	17.5	1,047,807	604,836
PVB_04.16	*P. simium*	*H. sapiens*	Brazil	84.1	61.3	37.0	6.1	7	10.5	1,033,576	546,833
PVB_05.17	*P. simium*	*H. sapiens*	Brazil	76.2	41.3	21.8	4.4	3	8.3	916,740	349,563
PVB_08.19	*P. simium*	*H. sapiens*	Brazil	73.6	35.7	17.5	2.9	3	6.2	893,168	304,391
PVB_24	*P. simium*	*H. sapiens*	Brazil	88.4	82.2	72.0	32.7	19	28.4	1,092,202	754,743
PVB_26	*P. simium*	*H. sapiens*	Brazil	81.1	51.7	29.1	5.6	5	10.1	997,937	451,490

*Note:* For each sample, the species, host, percentage of the genome covered with different minimum depths (≥ 1×, ≥ 5×, ≥ 10×, and ≥ 30×), median and mean coverage, and the number of sites retained in the two datasets (*dataset_GL* and *dataset_HF*) are shown. (see Figure S1 for the detailed pipeline and filtering procedure and Table S1 for the summary of sequencing coverage and single nucleotide variant sites included for all the samples analyzed in this study).

^a^ *P. vivax* sample from Brazil identified with significant admixed ancestry with the Ethiopian *P. vivax* population.

### 2.2 Genetic Relationships Among the *P. vivax* From Neotropical Non‐Human Primates, *P. simium,* and Worldwide Human *P. vivax* Populations

After filtering out multi‐strain infections and closely related isolates (see Materials and Methods), the final dataset included: 404 human *P. vivax* samples from 15 countries, four *P. vivax* from NHPs (two from Colombian *A. hybridus* , one from Colombian *A. seniculus* and one from Brazilian *Callicebus nigrifrons* ), and 19 *P. simium* isolates (*n* = 15 from *Homo sapiens* and *n* = 4 from *Alouatta guariba* , all from Brazil). Two additional *P. vivax* ‐like genomes from Cameroon chimpanzees ( *Pan troglodytes ellioti* ) (Loy et al. 2018) were included as outgroups (Figure 1). From this dataset, we created two datasets with different filtering strategies to accommodate the requirements of different population genetic analyses (Figure S1). The first dataset (*dataset_GL*, with _GL standing for Genotype Likelihood) was composed of 1,286,079 SNPs and all 429 genomes. This dataset_GL was designed for analyses accounting for genotype uncertainty through the use of genotype likelihoods rather than the genotype calls themselves using the ANGSD software ecosystem (Korneliussen et al. 2014), while permitting more tolerant filters, yet without introducing bias in genotype calls toward reference (see Korneliussen et al. (2014), Ros‐Freixedes et al. (2018) and Figure S2). In this dataset, *P. simium* samples exhibited an average number of 809,965 SNPs per sample (median: 916,740; standard deviation (sd): 249,332.68; range: 27,7549–1,101,940) and *P. vivax* samples from humans had an average number of 1,027,627 SNPs (median: 1,105,360; sd: 159,810.78; range: 214,273–1,109,456). The American *P. vivax* samples from NHPs presented 31,677 SNPs on average (median: 19,807; sd: 230,720.56; range: 5641–476,282). This framework allowed handling the large heterogeneity in average sequencing depth (mean: 88.86×; median: 63.6×, sd: 117.27; range: 2.5×–1142.5×), especially for the *P. vivax* isolates from Colombian NHPs, which had low genome coverage (Table 1).

The second dataset (*dataset_HF*, with _HF standing for Hard Filtering) relied on more classic SNP calling using a stringent filtering procedure (see Materials and Methods). It consisted of 819,652 high quality SNPs for 426 samples, excluding the three Colombian *P. vivax* isolates from NHPs due to their very low sequencing depth (less than 15% of genome ≥ 1×) and the large amount of missing data (> 90%) (Figure S1, Table S1 and Material and Methods). In this *dataset_HF*, *P. simium* samples exhibited an average number of 408,268 high quality SNPs per sample (median: 376,932; sd: 236,902.58; range: 67,372–809,449). In comparison, *P. vivax* samples from humans had an average number of 703,196 SNPs (median: 769,701; sd: 152,728.37; range: 636–819,247). The American *P. vivax* sample isolated from a monkey (sample P160) was covered by 38,682 SNPs (Table 1).

**FIGURE 2:**
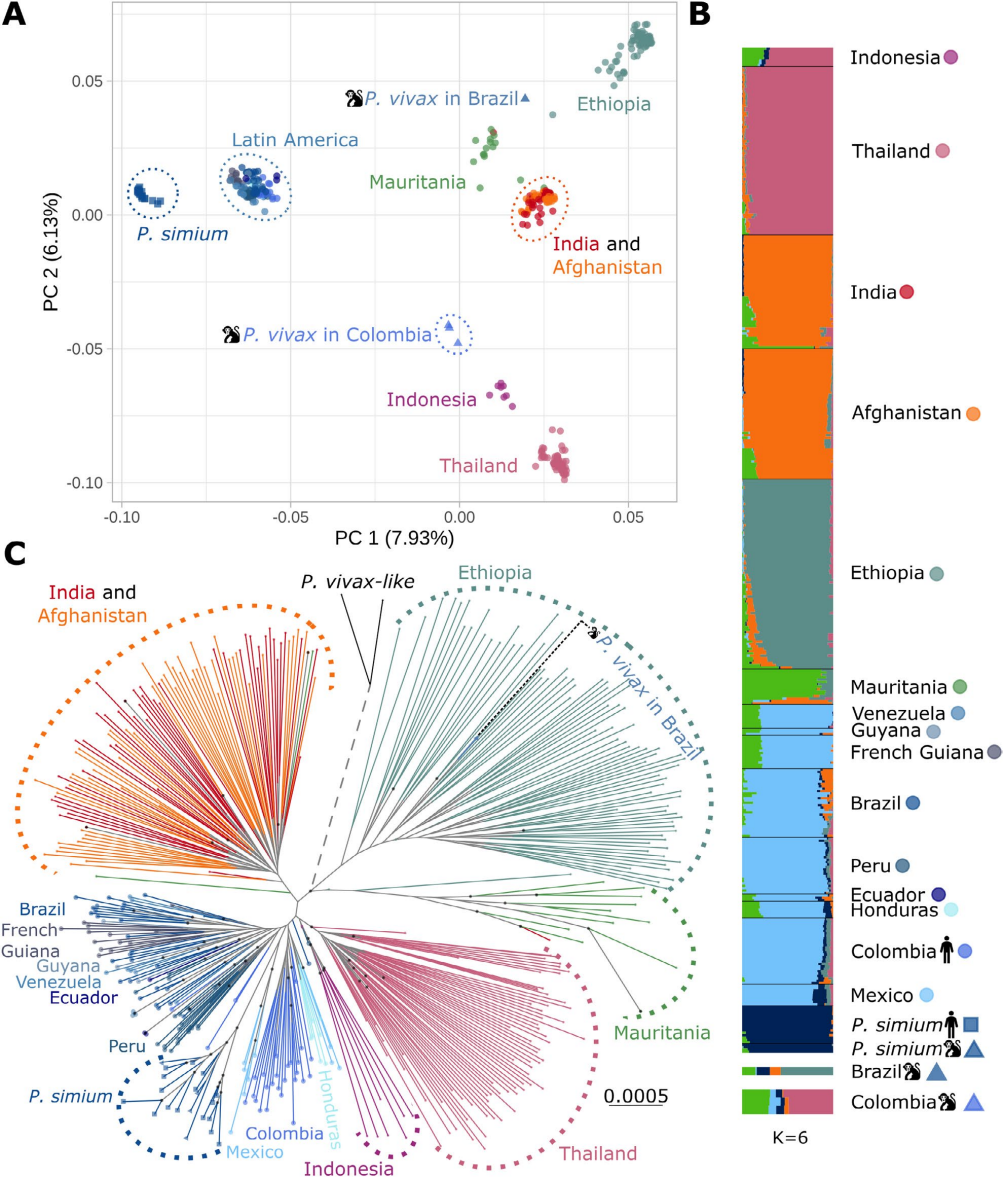
*P. vivax* and *P. simium* genetic structure. (A) Principal component (PC) analysis of 408 *P. vivax* and 19 *P. simium* strains showing the first and second PCs based on the genotype likelihood of 247,890 unlinked SNPs. (B) Individual genetic ancestry assuming *K* = 6 genetic clusters estimated using *PCAngsd* (see Figure S3 for the other K values). The monkey pictogram indicates the *P. vivax* samples from South American non‐human primates (NHPs). (C) Maximum likelihood phylogenetic tree of the 404 *P. vivax* , 19 *P. simium* and 2 *P. vivax‐like* individuals. *P. simium* and *P. vivax* from NHPs are indicated by triangles. The *P. vivax* isolate from a Brazilian NHP is among the Ethiopian *P. vivax* isolates. The three *P. vivax* samples from Colombian NHPs could not be included in this analysis because of the high number of missing data. The tree includes two *P. vivax‐like* strains used as outgroups. Note that the length of the outgroup branch (dotted lines) has been truncated. Black dots at nodes indicate highly supported nodes (both SH‐aLRT ≥ 80% and UFboot ≥ 95%, following the thresholds by Guindon et al. (2010) and Minh et al. (2013)).

Using these datasets, we first explored the genetic relationships of the simian *P. vivax* and *P. simium* isolates within the global genetic diversity of human *P. vivax* populations using multiple approaches. First, we performed a principal component analysis (PCA) and a model‐based individual ancestry analysis using the *dataset_GL* and *PCAngsd* (Meisner and Albrechtsen 2018) to assess the population structure. We also visualized genetic relationships among isolates using a maximum likelihood (ML) phylogenetic tree based on the *dataset_HF* with IQ‐TREE (Nguyen et al. 2015). The results were congruent (Figure 2) and revealed four distinct genetic clusters of human *P. vivax* , consistent with previous studies (Daron et al. 2021; Hupalo et al. 2016; Lefebvre et al. 2025): (1) East and Southeast Asia, (2) Africa, (3) Central Asia and South Asia, and (4) Latin America.

The four *P. vivax* isolates from NHPs did not cluster with *P. simium* isolates in the PCA (Figure 2A). The three Colombian simian *P. vivax* isolates were closely related to South‐East Asian *P. vivax* isolates, while the Brazilian isolate (P160) grouped together with African *P. vivax* , particularly Ethiopian samples (Figure 2A). The Colombian samples displayed admixed genetic ancestry (Figure 2B), with contributions from South‐East Asian and West African *P. vivax* populations. Conversely, the Brazilian sample shared genetic ancestry with *P. simium* and predominantly with Ethiopian *P. vivax* (Figure 2B). This isolate clustered within the Ethiopian diversity in the ML tree, although the node support was not high (SH‐aLRT = 95% and UFboot = 91%, lower than the thresholds established by Guindon et al. (2010) and Minh et al. (2013)) (Figure 2C).

In the PCA plot (Figure 2A), *P. simium* strains, isolated from NHPs and humans, formed a distinct cluster, separated from human *P. vivax* along the first principal component (PC 1). *P. simium* isolates were genetically closer to American *P. vivax* populations than to other populations from the rest of the world. The genetic ancestry plots further supported this result. Indeed, *P. simium* clustered with the American *P. vivax* when testing several numbers of clusters (*K*) ranging from 2 to 4 (Figure S3). Only with *K* = 5 did *P. simium* start to differentiate (Figure S3), although there was shared genetic ancestry, especially with *P. vivax* from Mexico and Honduras (dark blue in Figure 2B). The ML tree supported this result: *P. simium* branched within the genetic diversity of Mexican *P. vivax* , despite limited node support (SH‐aLRT = 100% and UFboot = 80%, lower than the thresholds established by Guindon et al. (2010) and Minh et al. (2013)).

**FIGURE 3:**
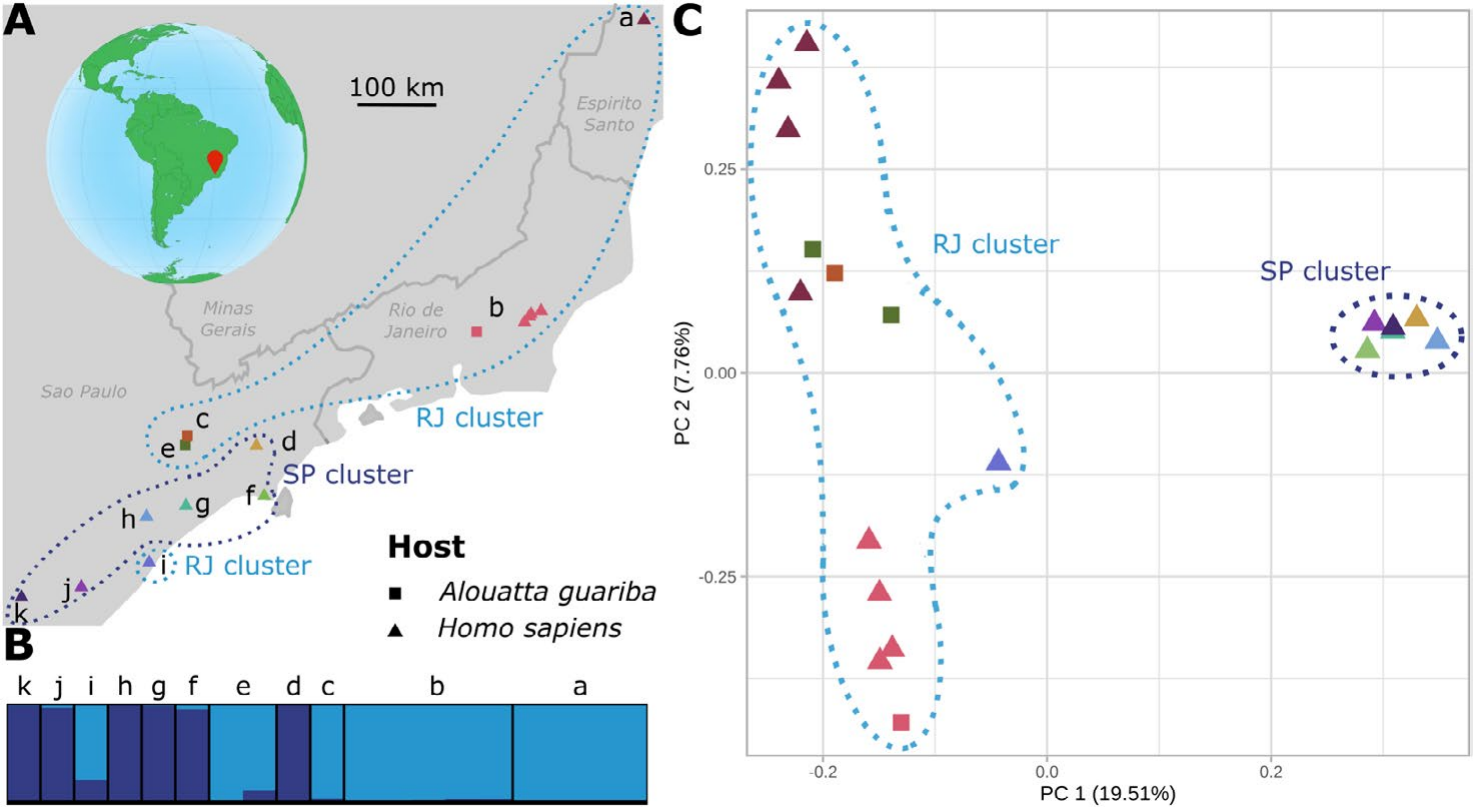
Geographic origins and genetic structure of the Brazilian *P. simium* isolates. (A) Geographic origins of the 19 *P. simium* isolates. The squares and triangles represent the host species (non‐human primates and humans, respectively), and the colors correspond to the region of origin. The SP cluster is outlined in dark blue, the RJ cluster in light blue. The borders and names of the Brazilian states are indicated in dark gray. On the globe, the red pointer indicates the region of Brazil from which the samples originated. Each site is designated by a letter: (a) Espírito Santo (*n* = 4), (b) Rio de Janeiro (*n* = 5), (c) São Paulo, Mairipora (*n* = 1), (d) Paraibuna (*n* = 1), (e) São Paulo, Cantareira State Park (*n* = 2), (f) Maresias (*n* = 1), (g) Riacho Grande (*n* = 1), (h) Juquitiba (*n* = 1), (i) Peruíbe (*n* = 1), (j) Registro (*n* = 1), (k) Iporanga (*n* = 1). (B) Individual genetic ancestry assuming *K* = 2 genetic clusters estimated using *PCAngsd* (see Figure S4 for the other *K* values). Each site is designated by a letter: (a) Espírito Santo (*n* = 4), (b) Rio de Janeiro (*n* = 5), (c) São Paulo, Mairipora (*n* = 1), (d) Paraibuna (*n* = 1), (e) São Paulo, Cantareira State Park (*n* = 2), (f) Maresias (*n* = 1), (g) Riacho Grande (*n* = 1), (h) Juquitiba (*n* = 1), (i) Peruíbe (*n* = 1), (j) Registro (*n* = 1), (k) Iporanga (*n* = 1). (C) Principal component (PC) analysis of 19 *P. simium* strains showing the first and second PCs based on the genotype likelihood of 44,911 unlinked SNPs. The shape (square and triangle) represents the host species (non‐human primates and humans), and the color the region of origin. The SP cluster is outlined in dark blue, the RJ cluster in light blue.

Although *P. simium* formed a well‐defined group distinct from the human *P. vivax* (Figure 2), the strains originated from various regions in Brazil and from different host species (humans and *A. guariba* ) (Figure 3A). Therefore, we investigated whether there was a genetic sub‐structuring within *P. simium*, independently of *P. vivax*, according to the host species and geography. The genetic ancestry analysis with *PCAngsd* (Meisner and Albrechtsen 2018) identified two genetic clusters within *P. simium* (Figure 3B, and Figure S4). This separation was also evident in the first principal component of the PCA (Figure 3C). The RJ cluster (*n* = 13) comprised *P. simium* samples from humans in the states of Rio de Janeiro and Espírito Santo, as well as in the municipality of Peruíbe, in the State of São Paulo. Additionally, *P. simium* samples from *A. guariba* from Rio de Janeiro and São Paulo were also included (Figure 3B). Within this cluster, the three samples from Rio de Janeiro, isolated from NHPs and humans, were distinct from the other samples in the RJ cluster along the second principal component of the PCA (Figure 3C). In contrast, the SP cluster (*n* = 6), located further south, included only human samples from the municipalities of Maresias, Riacho Grande, Juquitiba, Registro, and Iporanga, all within the state of São Paulo. Additionally, a sample from the RJ cluster (originating from Peruíbe) appears to exhibit admixture with the SP cluster, likely due to its geographical proximity (Figure 3B).

### 2.3 Evidence of Admixture History Between *P. simium* and *P. vivax*

We next investigated the role of admixture in shaping the genetic makeup of different populations and samples. We estimated and visualized the pairwise identity‐by‐descent (IBD) as a network depicting recent common ancestry among samples (Csardi and Nepusz 2006; Schaffner et al. 2018) (see the Materials and Methods). We also constructed population graphs using *AdmixtureBayes* and *TreeMix* (Nielsen et al. 2023; Pickrell and Pritchard 2012), to draw information on population branching order and potential admixture events (i.e., reticulations). We complemented these analyses with the *f*_*4*_ *‐statistics* which formally test for admixture among populations (Maier and Patterson 2024; Patterson et al. 2012). All these analyses were based on the *dataset_HF* (Figure S1).

**FIGURE 4:**
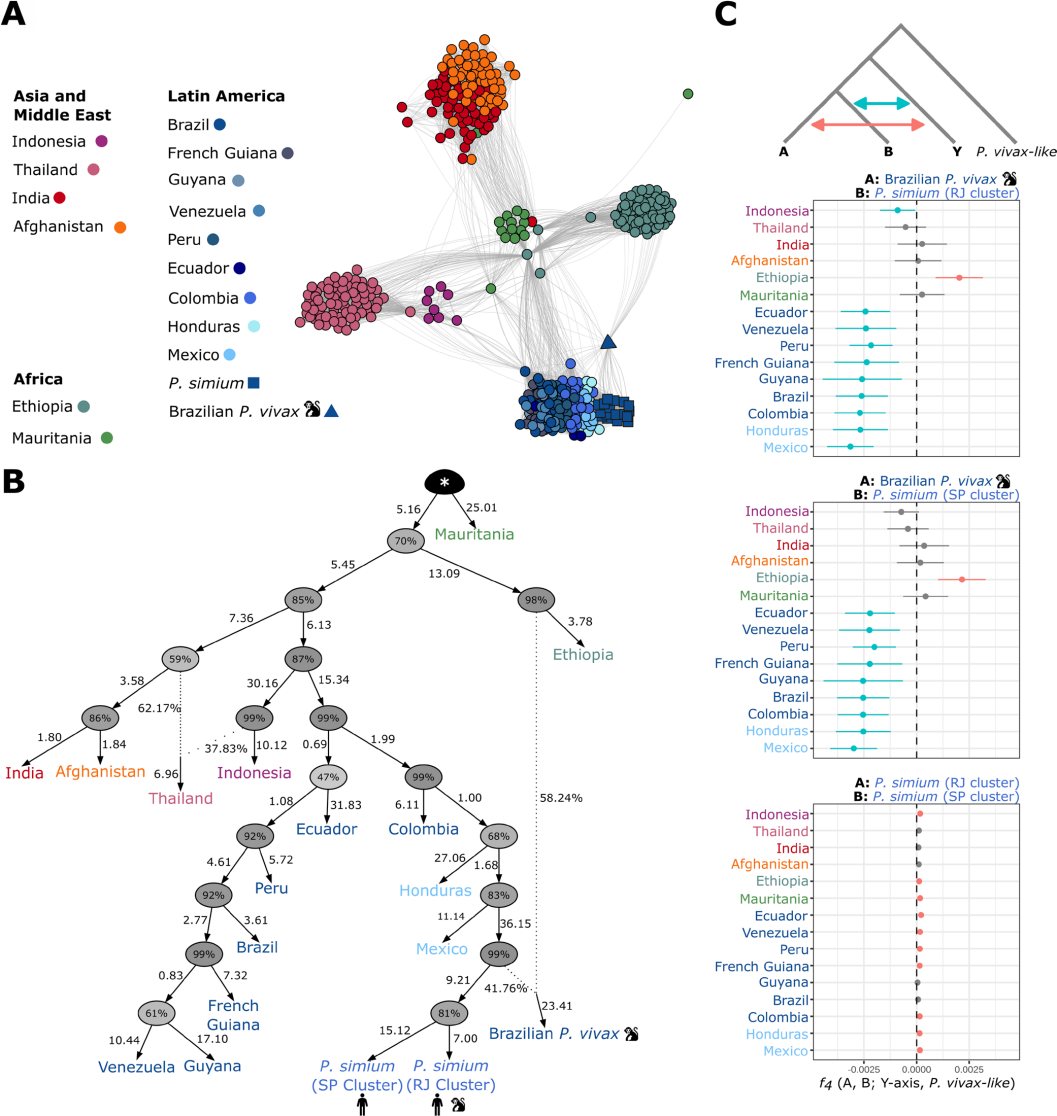
Population genetic relationships and admixture between *P. vivax* and *P. simium*. (A) Network visualization of the identity‐by‐descent (IBD) relationships among *P. vivax* and *P. simium* sample pairs. Edges represent the IBD values between each sample pair. Only edges with IBD values > 5% are displayed. (B) Admixture population graph topology of *P. vivax* and *P. simium* populations. The highest posterior probability was estimated with *AdmixtureBayes* (Nielsen et al. 2023), and rooted with the two *P. vivax‐like* genomes (*). The branch length (measured in drift units) indicates the genetic divergence between populations, multiplied by 100. Percentages at nodes indicate the posterior probability that the true graph has a node with the same descendants. For each admixture event (indicated by the dotted lines), the percentages show the admixture proportions from each donor. (C) Admixture tests based on the *f*_*4*_ *‐statistics* for *P. simium* clusters and the Brazilian *P. vivax* sample from a non‐human primate with *P. vivax* populations. This statistic formally assesses the excess of shared alleles between populations. In the form *f*_*4*_(A, B; Y‐axis, *P. vivax* ‐like), values not significantly different from 0 (gray) indicate no gene flow between populations A or B and the Y‐axis population. Positive values (red) suggest significant excess of allele sharing, and thus admixture or gene flow, between population A and the Y‐axis population, and negative values (blue) indicate admixture or gene flow between population B and the Y‐axis population. The monkey pictogram indicates the *P. vivax* isolate (P160) from a Brazilian non‐human primate.

As observed in the population structure analyses (Figure 2), the pairwise IBD network showed the same four main genetic clusters identified in *P. vivax* : Southeast Asia, Central and South Asia, East Africa, and Latin America. In the IBD network, all *P. simium* isolates formed a single coherent and genetically distinct cluster, closely related to Latin American *P. vivax* (Figure 4A). Conversely, the Brazilian simian *P. vivax* (P160) isolate was genetically distinct, showing strong connections with Ethiopian *P. vivax* and *P. simium* isolates, further underlining an admixed genetic ancestry as observed in previous analyses (Figure 2).

The *AdmixtureBayes* analysis identified a population graph with two admixture events as the best‐fitting solution for our data (Figure 4B and Figure S5). *P. simium* isolates formed a well‐supported monophyletic group with a common ancestor (posterior support > 80%) that connects both *P. simium* and the *P. vivax* population from Mexico (Figure 4B). The first reticulation in the *AdmixtureBayes* population graph identified Thailand *P. vivax* as an admixed population between Indonesian populations and the common ancestor of the Indian and Afghan populations. This reticulation was not visible in the pairwise IBD network, which only showed recent ancestry, and in *TreeMix*, which identified only one optimal migration edge (Figure S6). The only reticulation detected by *TreeMix*, also present in the *AdmixtureBayes* population graph, suggested that the Brazilian simian *P. vivax* isolate (P160) resulted from an admixture event between an unsampled East African *P. vivax* population closely related to the Ethiopian *P. vivax* and *P. simium*, with ~40% of its ancestry from *P. simium* (Figure 4B and Figure S6). These results were also supported by the *f*_*4*_‐statistic tests (Figure 4C), confirming that this simian *P. vivax* (P160) sample displayed a robust and consistent signal of genetically admixed ancestry between an East African population closely related to the Ethiopian *P. vivax* and the *P. simium* cluster.

Previous studies have shown that low coverage, when analyzed with traditional SNP‐calling approaches, may bias population structure analyses and generate spurious admixture signals (Johnson and Slatkin 2008; Ros‐Freixedes et al. 2018). In our study, we employed a statistical framework based on genotype likelihoods, specifically designed to reduce such biases (Korneliussen et al. 2014; Ros‐Freixedes et al. 2018). Moreover, all the samples retained more than 5000 SNPs, a threshold previously applied in population structure studies of ancient low‐coverage *P. vivax* (Michel et al. 2024). Nevertheless, we further evaluated the potential impact of low coverage by downsampling four high‐quality *P. vivax* genomes (one per distinct major genetic cluster) to an average coverage of 1.32× (comparable to the simian Colombian samples, see Table 1) with 100 replicates, and re‐estimated the PCA. Downsampling had only a minor effect on PCA results relying on the genotype likelihoods (Figure S2) and did not reveal any bias linked to the Asian origin of the reference genome (Auburn et al. 2016). This is consistent with the known robustness to this bias of genotype likelihood approaches compared to standard SNP‐calling pipelines (Korneliussen et al. 2014; Lou et al. 2021; Ros‐Freixedes et al. 2018). These results provide confidence in the genetic ancestry estimated from the PCA and PCA‐based admixture analyses for the Brazilian P160 and the three simian Colombian samples. The other analyses based on the *dataset_HF* (ML tree, IBD network, *AdmixtureGraph*, TreeMix, *f4‐statistics*) only included one low‐coverage sample, P160. Although the pipeline for *dataset_HF* is more stringent and is a commonly used approach for producing high quality SNPs (Daron et al. 2021; Lefebvre et al. 2023, 2025), it delivered nearly 39,000 high quality SNPs for P160 (Table 1). Finally, the admixed genetic ancestry estimates for the P160 sample obtained from the two statistical frameworks (based on the GL and HF datasets) were fully consistent with each other (Figures 2 and 4). Altogether, our analytical pipeline and robustness analyses suggest that the admixture signal detected in the four low‐coverage samples (P160 and the three Colombian isolates) is a genuine signal, and did not reflect an artefact of lower sample quality, lower coverage, or a potential reference bias.

### 2.4 *Plasmodium simium* Demographic History

**FIGURE 5:**
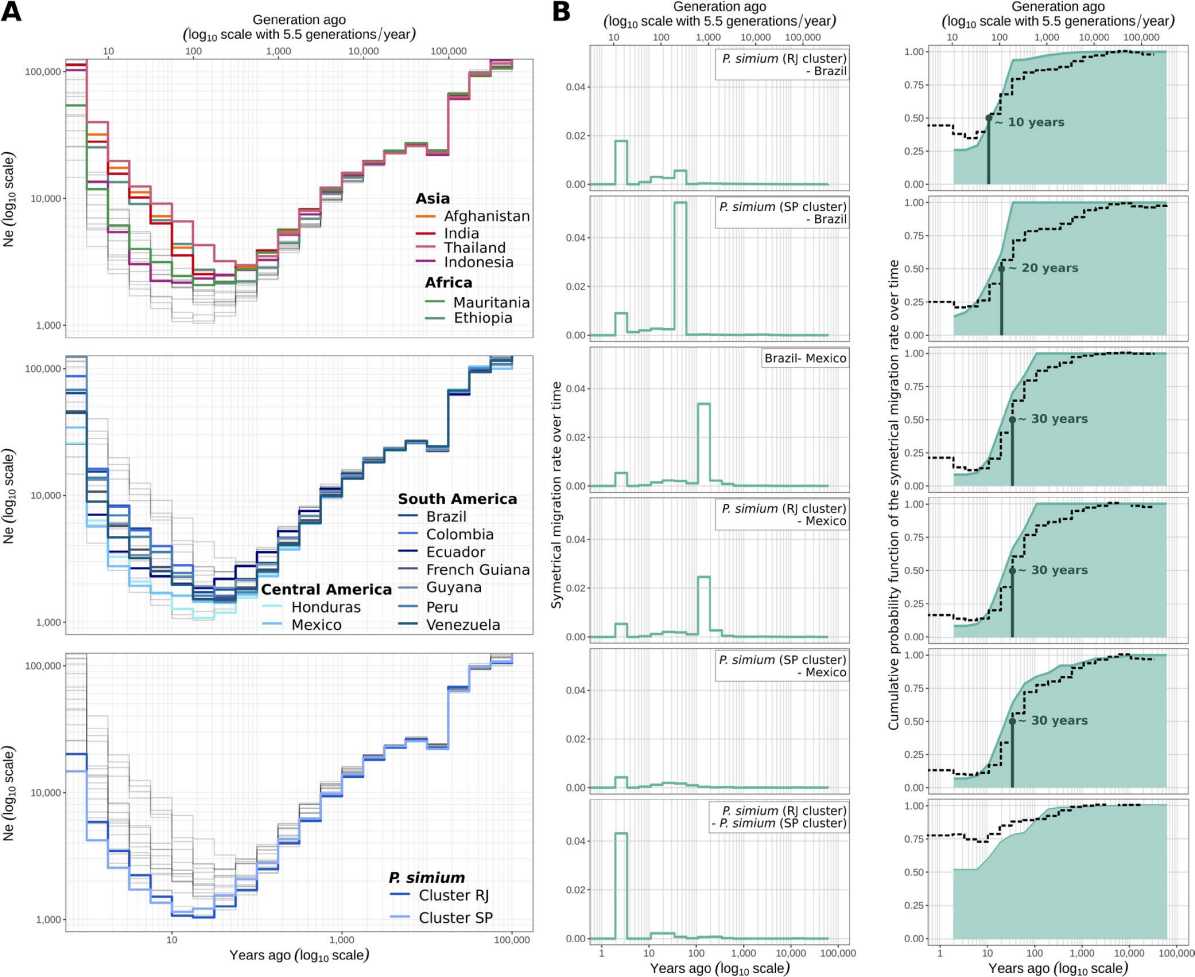
Coalescent‐based inference of the demographic history and migration rates of *P. vivax* and *P. simium* populations. (A) The variation in effective population (*Ne*) size was estimated using *Relate* (axes are log_10_ transformed). (B) Timing and dynamics of the separation between the indicated groups estimated using *Relate* coalescence rates and *MSMC‐IM*. On the left, the time‐dependent symmetric migration rate (x‐axes are log_10_ transformed). On the right, the cumulative distribution probabilities of the migration rates as a function of time. This is an estimate of the proportion of ancestry already merged at time *t*, and represents the proportions of gene flow over time (x axes are log_10_ transformed). Values close to 0 indicate complete isolation between groups, while 1 shows a complete mixture as one population. Dashed lines indicate the relative cross‐coalescence rate. The divergence time (when the cross‐coalescent rate drops below 50%) is specified in each panel.

We further investigated *P. simium* demographic history, potential founder effect, and isolation dynamics. We first computed the genome‐wide distribution of the nucleotide diversity (*π*) in windows along the core genome of populations with at least 10 samples. *P. simium* exhibited significantly lower *π* values than *P. vivax* (*p‐value* < 0.001, Wilcoxon signed‐rank test with Bonferroni correction, Figure S7). Then, we explored the changes in effective population size (*N*_*e*_) over time, estimated using coalescence rates with *Relate* (Speidel et al. 2019). In agreement with the *π* values (Figure S6), both *P. vivax* and *P. simium* populations worldwide exhibited a steady decline in *N*_*e*_ starting ~100,000 generations ago or ~20,000 years ago (assuming a generation time of 5.5 generations/year and a mutation rate of 6.43 × 10^−9^ mutations/site/generation (Daron et al. 2021)), followed by a recent moderate expansion (Figure 5A). Notably, the two *P. simium* clusters (RJ and SP) displayed lower *N*_*e*_ values than the *P. vivax* populations, although we did not detect any strong signal of bottleneck associated with a potential founder effect.

To explore the isolation and migration history of *P. simium* from *P. vivax* in Latin America, we used Multiple Sequentially Markovian Coalescent‐Isolation Migration program (MSMC‐IM) (Wang et al. 2020) to estimate the isolation dynamics and the migration rate changes over time. Our results, based on the cross‐coalescent rates (CCR) between *P. simium*, Brazilian, and Mexican *P. vivax* populations, suggested a relatively recent isolation of *P. simium* from *P. vivax* . Based on the point at which the CCR dropped below 50% as suggested by Wang et al. (2020), we could estimate that in all pairwise comparisons between *P. simium* and Brazilian/Mexican *P. vivax* , split times occurred between 10 and 30 years ago (Figure 5). The only exception was between the two *P. simium* clusters, where CCR values remained at ~75%, indicating that genetic exchange was still ongoing (Figure 5B, bottom).

Migration rate estimations (Figure 5B) suggested that the oldest migration events occurred 100–200 years ago between the Mexican and Brazilian *P. vivax* populations, and between Mexican *P. vivax* and *P. simium* from the RJ cluster. The estimated divergence times were ~30 years ago for both the Brazilian‐Mexican *P. vivax* and Mexican *P. vivax* ‐*P. simium* RJ cluster. Similarly, the divergence time between the *P. simium* SP cluster and Mexican *P. vivax* was ~30 years ago, with minimal genetic exchange over the entire period (Figure 5B). Conversely, the *P. simium* SP cluster and Brazilian *P. vivax* populations exchanged genetic material more recently (30–60 years ago), with divergence estimated at ~20 years ago. On the other hand, the RJ cluster showed limited recent exchanges with Brazilian *P. vivax* , and diverged ~10 years ago. Importantly, although the cumulative migration probability (*Mt*), which is analogous to the CCR (Schiffels and Durbin 2014; Wang et al. 2020), was close to zero for some comparisons between *P. vivax* and *P. simium*, none of them reached zero. This suggests that genetic exchanges may still be possible, even at a low rate (Figure 5B).

### 2.5 Genetic Evidences of *P. simium* Adaptation to New Hosts

**FIGURE 6:**
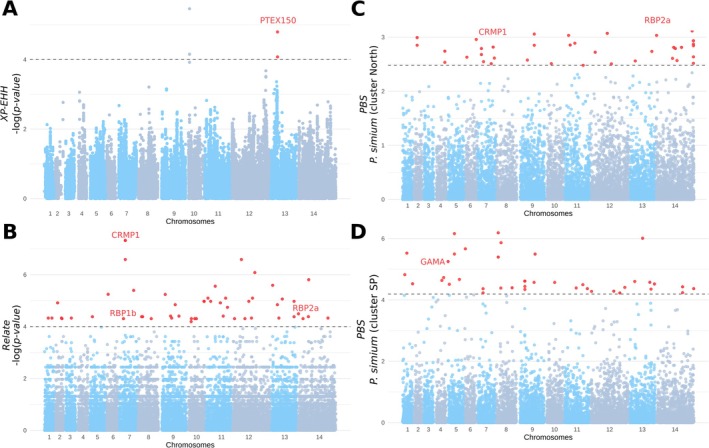
Evidence of selective sweeps in *P. simium*. (A) Manhattan plot showing the haplotype‐based *XP‐EHH* statistics between pairs of populations. The dotted line represents the significance threshold value at –log (*p‐value*) = 4. Red points are the candidate SNPs identified as potentially affected by a selective sweep in *P. simium* (negative *XP‐EHH* values). (B) Manhattan plot showing the *Relate* results. The dotted line represents the threshold significance value –log (*p‐value*) = 4. The red points are the candidate SNPs identified as potentially affected by positive selection in *P. simium*, based on their higher‐than‐expected coalescence rate compared with the rest of the genome. (C and D) Manhattan plots showing the *PBS* values, an *F*_*ST*_‐like statistic that captures a difference in allele frequencies compared with two reference populations, for each *P. simium* cluster, with *P. vivax* Brazilian and Colombian samples as outgroup populations. For the *PBS* scores, all values in red represent the top 0.1% of the PBS values, suggesting higher allelic frequency differentiation compared to the reference populations, and thus indicating significant evidence of positive selection that affects these SNPs. CRMP1, cysteine repeat modular protein 1; RBP1b, reticulocyte binding protein 1b; RBP2a, reticulocyte binding protein 2a; GAMA, GPI‐anchored micronemal antigen.

The host jump from humans to NHPs likely exerted strong selective pressure on *P. simium*, potentially leaving detectable signals of positive selection in its genome. Therefore, we first used haplotype‐based tests within (*iHS* (Voight et al. 2006)) and between (*XP‐EHH*, and *Rsb* (Sabeti et al. 2007; Tang et al. 2007)) populations to identify signals of positive selection specific to a target population. These tests use haplotype length variations (i.e., linkage disequilibrium, LD) to detect recent or ongoing selective events (Sabeti et al. 2007; Tang et al. 2007; Voight et al. 2006). Given the limited sample size (*n* = 19), we treated the two *P. simium* clusters as a single group and compared it to the Brazilian *P. vivax* population as the reference population for *XP‐EHH* and *Rsb* statistics. The datasets derived from *dataset_HF*, following the determination of ancestral and derived alleles with *P. vivax‐like* genomes, included 10,304 SNPs for the 19 *P. simium* samples for the *iHS* test, and 378,441 SNPs for 48 samples (*n* = 19 *P. simium* and *n* = 29 Brazilian *P. vivax*) for the *XP‐EHH* and *Rsb* analyses (see Material and Methods for more details). The within‐population *iHS* test identified a single significant SNP within the coding sequence (CDS) of the *PVP01_1139100* gene, an orthologue of a *Plasmodium* protein of unknown function (Figure S8). The *XP‐EHH* and *Rsb* analyses identified two significant SNPs in the CDS of the *PTEX150* gene (Figure 6A, Figure S8 and Table S3), which encodes a protein involved in protein transport across the parasitophorous vacuolar membrane during the *Plasmodium* erythrocytic cycle (Beck and Ho 2021; Elsworth et al. 2016).

The haplotype‐based tests may miss other genomic regions because recombination can break down LD patterns. Therefore, we used *Relate* (Speidel et al. 2019) to detect positive selection by identifying increased coalescence rates in selected regions compared with the rest of the genome. By analyzing 26,565 SNPs in the 19 *P. simium* samples, we identified 49 SNPs with significant signals of selection in the CDS of 14 genes (Table S3). Notably, three of these genes are involved in the interactions of the parasite with primate hosts (*PvRBP2a* and *PvRBP1b* (Galinski et al. 1992, 2000; Galinski and Barnwell 1996)) and mosquitoes (*PvCMRP1* (Hart et al. 2014; Kenthirapalan et al. 2016; Thompson et al. 2007)).

We also investigated cluster‐specific evidence of adaptation in *P. simium* by calculating the population branch statistic (*PBS*), an *F*_*ST*_‐like statistic, by comparing each *P. simium* cluster with two American *P. vivax* populations (Colombia and Brazil). Outlier values (top 0.1% of the 41,338 1‐kb sliding windows for the RJ cluster and of the 39,799 1‐kb sliding windows for the SP cluster) indicated significant differentiation of the *P. simium* clusters compared with American *P. vivax* . We identified 43 outlier windows in the RJ cluster and 41 in the SP cluster, corresponding to positive selection in the CDS of 32 genes (RJ cluster) and 31 genes (SP cluster) (Table S3). Four genes showed signs of positive selection in both clusters (*PVP01_0525300*, *PVP01_0931400*, *SMC1*, and *PVP01_1469500*). *SMC1* (Structural Maintenance of Chromosomes protein 1) is involved in the structural maintenance of chromosomes in eukaryotes (Losada et al. 1998; Yoshinaga and Inagaki 2021). Conversely, the functions of the other three genes are unknown. In the RJ cluster, two genes with evidence of selection were also detected with *Relate*: *PvRBP2a* and *PvCMRP1*. These play a direct role in the interactions with the primate hosts and with mosquitoes, respectively. The SP cluster showed selection signals in *PvGAMA*, a gene involved in binding to human erythrocytes (Cheng et al. 2016).

In conclusion, we identified 73 genes under positive selection in *P. simium*, including several implicated in interactions with both mosquito vectors and primate hosts.

## 3 Discussion

Historically, natural *P. vivax* or *P. simium* infections in NHPs in Brazil were detected with antigenic tests and PCR assays (Costa et al. 2014; de Castro Duarte et al. 2008), although the two species were often indistinguishable. *Plasmodium simium* was long thought to circulate exclusively in Brazil (Alvarenga et al. 2017; Brasil et al. 2017; Costa et al. 2014), but recent reports of natural infections in Colombian and Costa Rican NHPs, attributed to *P. vivax* or *P. simium* (Chaves et al. 2022; Rondón et al. 2019), questioned the geographic distribution of *P. simium*. By screening 719 NHPs samples from five different Latin American countries, we show that *P. simium* remains restricted to Brazil, while *P. vivax* naturally infects at least two Neotropical NHP species ( *A. hybridus* and *A. seniculus* ) in other parts of Latin America. Importantly, all available *P. simium* genomes analyzed to date originate from southeastern Brazil (Rio de Janeiro and São Paulo), and conclusions regarding its evolutionary history and geographic range should therefore be interpreted within this restricted sampling framework. These findings suggest ongoing human to NHP transmission and highlight the reverse zoonotic potential of these parasites as already suggested (de Oliveira, Rodrigues, Duarte, et al. 2021).

The three Colombian simian *P. vivax* samples were genetically closer to South‐East Asian than American *P. vivax* populations (Figure 2), an unexpected pattern not observed in human American isolates (Daron et al. 2021; Hupalo et al. 2016; Kattenberg et al. 2024; Lefebvre et al. 2025). Importantly, this genetic proximity does not imply recent or direct transmission from Southeast Asia, but may reflect unsampled diversity or complex historical population structure. Sensitivity analyses confirmed that this pattern is genuine and not driven by an artefact of lower sample quality, lower coverage, or reference bias (Figure S2). Given the very limited number of simian *P. vivax* genomes currently available and their heterogeneous sequencing depth, interpretations regarding their evolutionary origin remain speculative. Additional sampling of NHPs in Colombia and neighboring regions will be required before robust conclusions can be drawn regarding the origin of these infections.

Reanalysis of the Brazilian NHP isolate P160 using genotype‐likelihood approaches revealed a robust signal of admixture between *P. simium* and an East African *P. vivax* population (Figures 2B and 3), consistent with previous observations (de Oliveira, Rodrigues, Duarte, et al. 2021). This framework enabled us to recover a substantially larger fraction of the genomic variation (> 45% of the core genome, (Table 1)), allowing a more reliable inference of the sample genetic ancestry. Although this admixture signal is robust across analytical frameworks, it is based on a single sample. Consequently, any historical interpretation regarding its origin, including the potential involvement of imported African strains, should be considered preliminary. Broader sampling of NHPs and humans in Brazil will be necessary to determine whether this represents an isolated event or part of a wider transmission process. More generally, the close genetic proximity between *P. simium* and *P. vivax* is consistent with a relatively recent host jump (~80–100 years ago) and ongoing gene flow detected between these taxa (Figure 5B). Interestingly, the genetic ancestry of P160 was predominantly (~60%) of Ethiopian origin (or a closely related unsampled population), and the rest from *P. simium* (Figure 4B). While unexpected given the Brazilian origin of the sample, this observation cannot currently be placed into a broader evolutionary context due to the absence of additional samples with similar ancestry.

The discovery of natural *P. vivax* infections in Colombian NHPs suggests that NHPs may act as reservoirs, potentially complicating malaria elimination efforts in Latin America and affecting NHPs health and conservation efforts. *Plasmodium vivax* remarkable adaptability contrasts with *Plasmodium* species of the subgenus *Laverania* in which host transfers are rarely observed (Su and Wu 2021). Ongoing genetic exchanges between *P. vivax* and *P. simium* further indicate porous species boundaries and continued recombination. Together, these findings underscore the need to consider NHPs in malaria control strategies, while also accounting for the conservation of already threatened NHPs (IUCN 2024), particularly in light of past culling events that have had negative consequences for both wildlife conservation and public health (Bicca‐Marques and de Freitas 2010).

The evolutionary processes underlying *P. simium* host shift and adaptation have not been fully explored so far (but see de Oliveira, Rodrigues, Duarte, et al. (2021)). Using multiple complementary population genomic approaches, we consistently recovered a genetic structure among *P. vivax* populations (Figure 2, Figure 4A,B) that is in line with previous findings (Daron et al. 2021; Hupalo et al. 2016; Lefebvre et al. 2025). *P. simium* formed a distinct cluster closely related to American *P. vivax* , particularly Mexican strains (Figures 2C and 4B, Figure S6), as previously reported (de Oliveira, Rodrigues, Duarte, et al. 2021; Mourier et al. 2021). Our results, supported by the ancestry plot (Figure 2B), the population graph, and the *f*_*4*_‐statistics (Figure 4), showed no genetic evidence for an Asian contribution to *P. simium* genetic ancestry, in contrast to early studies based on mitochondrial DNA (Carter 2003; Li et al. 2001), supporting an American origin for *P. simium*, closely linked to Central or North American *P. vivax* . Alternative scenarios (including unsampled American source populations or multiple independent introductions from Latin America) cannot be excluded given current sampling limitations. Nevertheless, in the absence of additional data, a North/Central American origin remains the most parsimonious explanation, supported by concordant signals across multiple population genomic analyses and by the absence of detectable Asian or African ancestry. Although *P. simium* is currently restricted to Brazil, documented historical gene flow among American *P. vivax* populations (Daron et al. 2021; Kattenberg et al. 2024; Lefebvre et al. 2025), likely explains the retention of a more pronounced “North/Central American‐like” ancestry in *P. simium* compared with Brazilian *P. vivax* .

A finer‐scale analysis of *P. simium* genetic structure revealed two distinct genetic clusters (Figure 3): RJ and SP. The SP cluster contains only human isolates, whereas the RJ cluster included both *A. guariba* and human samples. This previously unreported structure likely reflects improved sampling and data integration, but remains complex. Indeed, the PCA results indicate that samples from Rio de Janeiro city are genetically distinct within the RJ cluster, while the Peruíbe sample is geographically closer to the SP cluster, but its genetic ancestry is mainly from the RJ cluster. Additional genomic sampling across regions and host species will be required to resolve the evolutionary and epidemiological significance of this structure.

The close genetic similarity between human *P. vivax* and NHP *P. simium* suggests recent host transfer(s), as previously proposed (de Oliveira, Rodrigues, Early, et al. 2021; de Oliveira, Rodrigues, Duarte, et al. 2021; Mourier et al. 2021), but for which no precise timing had been estimated. Our coalescent‐based analyses estimate that *P. simium* diverged from Mexican/Brazilian *P. vivax* within the last 30 years (Figure 5B), with significant migration occurring ~100–200 years ago between Mexican and Brazilian *P. vivax* populations and between the *P. simium* RJ cluster and Mexican *P. vivax* (Figure 5B). These results suggest that a first host jump may have occurred ~100–200 years ago when some Central or North American *P. vivax* migrated to Brazil. Unfortunately, limited sampling from Mexico and neighboring regions prevents identification of the source population. Interestingly, the estimated divergence times overlap with several historical migration events in the Americas, including the migration of several thousand confederates from the Southern United States to Latin America, including Mexico and Brazil, at the end of the American Civil War (Hill 1935; Sutherland 1985). It is possible that *P. vivax* strains from the Southern United States, potentially genetically close to Mexican populations, may therefore represent one hypothetical source for the RJ cluster in *P. simium*. However, this remains only one hypothesis among others, and broader genomic sampling across North and Central Americas will be necessary to refine the evolutionary origin of *P. simium*.

The *P. simium* SP cluster showed no evidence of genetic exchanges with Mexican *P. vivax* , but exhibited gene flow with Brazilian *P. vivax* dating to ~30–60 years ago. *MSMC‐IM* analysis further indicated ongoing gene flow between the *P. simium* clusters, with CCR values well above 50% (threshold to suggest split time following (Wang et al. 2020)) and close to 75%, along with the high migration rate ~2 to 4 years ago, suggesting incomplete divergence and possible continued genetic exchange. Accordingly, the SP cluster origin remains unresolved. Population graphs linked both *P. simium* clusters connected to an ancestor closely related to Mexican *P. vivax* (Figure 5), while the *MSMC‐IM* results indicated a more recent origin of the SP cluster (30–60 years ago) from Brazilian human *P. vivax* (Figure 6). The close genetic proximity between the two *P. simium* clusters may reflect recent secondary contacts, as suggested by high recent migration rates (~2–4 years ago, see Figure 5B) and the presence of an admixed individual (Figure 3B). Nevertheless, the small sample size in the SP cluster (*n* = 6) prevents robust inference about its origin and evolutionary dynamics. Previous studies proposed the possibility of multiple host jumps based on the presence of polymorphisms in few mitochondrial or nuclear markers (Lim et al. 2005; Tazi and Ayala 2011). An alternative hypothesis could be a single host jump that involved several *P. vivax* strains infecting NHPs. Therefore, we cannot rule out the possibility that the SP cluster derived from the RJ cluster and that the genetic distinction observed between clusters might be attributed to host adaptation dynamics. Specifically, the RJ cluster infects both simian and human hosts, whereas the SP cluster seems to infect exclusively humans. Larger sampling and more geographically diverse will be needed to determine the role of host specificity, human movement, and demographic history. Furthermore, MSMC‐IM does not provide explicit confidence intervals, but split time is estimated from the drop of coalescent rates across populations below a certain threshold (50%). Therefore, the estimated divergence and migration times should be interpreted with caution. Coalescent‐based scenario‐testing methods, such as *DIYABC‐RF* (Collin et al. 2021), *dadi* (Gutenkunst et al. 2009) and *fastsimcoal* (Excoffier et al. 2021), could help clarify *P. simium* evolutionary history and the uncertainty in parameter estimations.

Importantly, the inferred divergence times may be biased by the life‐history traits of *P. simium* and *P. vivax* . *Relate* and *MSMC‐IM* rely on the Wright‐Fisher (WF) coalescent assumptions (Kingman's coalescent model (Kingman 1982)) that assume panmixia, non‐overlapping generations and neutral evolution (Fisher 1923; Wright 1931). These assumptions are unlikely to fully apply to *P. vivax* , and probably not for *P. simium*, which exhibit dormancy and complex life cycles (Imwong et al. 2007; White 2011). Such traits may maintain genetic diversity, attenuate the effect of genetic drift, and inflate the effective population size (Ferreira et al. 2014; Mwima et al. 2024), potentially explaining the absence of any detectable bottleneck signal in the *P. simium* population size during the host shift (Figure 5). Moreover, *Relate* and *MSMC‐IM* rely on Kingman's coalescent model which assumes one pairwise lineage coalescence per generation (Kingman 1982). This may be violated in *Plasmodium* parasites, whose life cycle alternates between asexual reproduction in primates and sexual reproduction in mosquitoes, potentially leading to simultaneous coalescent events (Tellier and Lemaire 2014). When such events are frequent, Kingman's model underestimates the effective population sizes, although it remains accurate if they are rare (Korfmann et al. 2024). To address these biases, we excluded clonal and closely related individuals (see Materials and Methods). Nevertheless, the complex biology of *P. simium* and *P. vivax* , combined with uncertainty in mutation rate and generation time, parameters known to vary spatially and temporally, likely affects the accuracy of divergence time and population size estimates. In this study, we used values commonly adopted in the literature (Daron et al. 2021; Lefebvre et al. 2025), which have been discussed as reasonable approximations for *P. vivax* (Lefebvre et al. 2025), but we acknowledge that these parameters remain difficult to define. Limited sampling of *P. simium* likely further contributes to this uncertainty and may explain why inferred divergence dates are more recent than the earliest historical descriptions of the parasite (Da Fonseca 1951; Deane et al. 1966; Mourier et al. 2021).

Through its evolution, *P. simium* has experienced both human‐to‐wild animal (reverse zoonosis) and wild animal‐to‐human (zoonosis) transmission, making it an interesting candidate for studying the genetic basis of host adaptation. The recent host shift has likely imposed significant selection pressure on *P. simium* genomes, and our results reveal signatures of selection acting on the standing genetic variation. Specifically, we found significant signals in two genes, *PvRBP2a* and *PvRBP1b* (Figure 6), which play key roles in reticulocyte invasion in the NHP hosts (Galinski et al. 1992, 2000; Galinski and Barnwell 1996). Previous studies identified a deletion in the *PvRBP2a* CDR region in *P. simium* (de Oliveira, Rodrigues, Duarte, et al. 2021; Mourier et al. 2021), and our findings suggest that these selection signals may reflect linked selection near the deletion (~1912 nucleotides away). Notably, this is the first report of positive selection in *PvRBP1b* in *P. simium*. Given the essential role of these genes in reticulocyte invasion (Galinski et al. 1992, 2000; Galinski and Barnwell 1996), our results suggest adaptive changes facilitating infection of new primate hosts. It would therefore be relevant to study the impact of this parasite on the health and survival of the NHPs in this region, which are already threatened (IUCN 2024). Noteworthy, *PvRBP1b* is also under selection in *P. vivax* (Benavente et al. 2021), and the signal we observe is detected solely through intra‐population tests and may represent a continuation of selection already present in *P. vivax* , rather than a *P. simium*‐specific adaptation. In addition, within the SP cluster, which infects only humans, we discovered significant divergence in the *PvGAMA* gene relative to Colombian/Brazilian human *P. vivax* (Figure 6D). Although *PvGAMA* binds to human erythrocytes irrespective of their Duffy antigen status (Cheng et al. 2016), this signal was detected only in the PBS genome scan (Figure 6D). Because PBS does not always distinguish true signals of positive selection from the effects of purifying selection, particularly in populations that have experienced bottlenecks (Shpak et al. 2025), its interpretation requires caution. Given that *P. vivax* is capable of dormancy (McGregor and Krotoski 1985), it is plausible that *P. simium* shares this trait, which could mitigate the impact of bottlenecks on genetic diversity (Ferreira et al. 2014) and allow us to observe genuine selection signals. Functional studies will be necessary to determine whether variation in PvGAMA contributes to host‐specific adaptation in *P. simium*.

*P. simium* adapted not only to primate hosts but also to new mosquito vectors. Unlike *P. vivax* , *P. simium* is thought to be confined to the Atlantic coast of Brazil and transmitted primarily by anopheline mosquitoes of the *Kerteszia* subgenus, mainly *An. cruzii* and *An. bellator* (Marrelli et al. 2007; Costa et al. 2014). We identified selection signals in the *PvCMRP1* gene across all *P. simium* samples and in *PvPAT* only within the SP cluster (Figure 6). Because previous studies showed that *PvCMRP1* underwent positive selection in Latin American *P. vivax* populations (Benavente et al. 2017, 2021; Hupalo et al. 2016), the signal observed in *P. simium* may reflect shared evolutionary pressure in American *P. vivax* . Although the exact functions of *PvCMRP1* and *PvPAT* remain unclear, functional studies of orthologs showed their crucial roles in the mosquito life cycle and transmission to vertebrate hosts (Hart et al. 2014; Kenthirapalan et al. 2016; Thompson et al. 2007).

de Oliveira, Rodrigues, Duarte, et al. (2021) detected selection signals in the *pvs47* gene (potentially essential for avoiding the mosquito immune system), the sequence of which differs between *P. vivax* and *P. simium* by only two amino acid substitutions (Tachibana et al. 2015). Although both SNPs are present in our dataset, we did not find them to be under selection. The small *P. simium* sample size (*n* = 19) may have limited the power of our analyses, particularly with methods like *Relate* and haplotype‐based tests. In addition, *PBS* genome‐scans, while more robust to small sample sizes (Willing et al. 2012), may have lacked power because of the similarity of *P. simium* sequences to those of Brazilian and Colombian *P. vivax* (de Oliveira, Rodrigues, Duarte, et al. 2021) used as the reference populations. Therefore, additional *P. simium* samples will be necessary to robustly identify such signals in *pvs47*.

We found no evidence of selection in *P. simium* genes associated with resistance to antimalarial treatments, primarily chloroquine and primaquine. In *P. vivax* , chloroquine resistance was not reported in Latin America before 2000 and remains incompletely characterized, and no in vivo resistance to primaquine has been documented (Ferreira et al. 2021; Price et al. 2014). Consequently, treatment resistance has not been reported in humans infected with *P. simium* (Brasil et al. 2017). While this may indicate a lack of resistance in *P. simium*, the limited sample size (*n* = 19) may have been insufficient to detect such signals.

Noteworthy, we detected significant positive selection signals in 73 genes, including *PvIMC1h*, *PvG377*, and *PvGRASP* (Table S3), which might be involved in adaptation to primate hosts and/or mosquito vectors. Although the functions of many of these genes remain unknown, functional validation will be necessary to confirm their role in *P. simium* adaptation. Given the limited *P. simium* sample size (*n* = 19), genes identified in selection scans should be considered putative candidates rather than definitive targets of selection, as genome‐wide analyses are expected to include some false positives despite multiple‐testing correction.

To conclude, zoonotic malaria is a significant public health concern in Brazil, as highlighted by a recent *P. simium* outbreak in the Atlantic Forest of Rio de Janeiro. This underscores the potential role of NHPs as malaria reservoirs and the need for extensive research on their genetic diversity, evolutionary history, and zoonotic potential, as well as the largely unexplored health and conservation impacts on endangered primate populations. Based on 719 NHP samples collected across five Latin American countries, we provide the first evidence of natural *P. vivax* infections in Colombian NHPs, highlighting the possibility of ongoing host switches and their relevance for malaria elimination strategies. The genetic analyses revealed recent admixture events and gene flow between *P. simium* and *P. vivax* , emphasizing the porous boundaries between these parasites. We further show that *P. simium* forms a distinct cluster closely related to American *P. vivax* populations and comprises at least two genetic clusters in southeastern Brazil. Despite limited sampling, we identified genes potentially involved in *P. simium* adaptation to NHPs, human red blood cells, and mosquito vectors. Our findings question the distinction between *P. simium* and *P. vivax* because both can infect humans and Neotropical NHPs and produce hybrids, suggesting that *P. simium* may represent distinct *P. vivax* populations. By highlighting the potential NHPs role as malaria reservoirs and the porous genetic boundaries between *P. vivax* and *P. simium*, our study underscores the need to incorporate zoonotic dynamics into malaria control strategies, while promoting coexistence between humans and NHPs. Although our analyses reveal clear evidence of host shifts and genetic exchange between *P. vivax* and *P. simium*, interpretations regarding the evolutionary history of *P. vivax* infections in non‐human primates remain limited by sample availability. Expanding genomic sampling of simian malaria parasites will be essential to fully resolve these dynamics.

## 4 Materials and Methods

### 4.1 *P. simium* Sample Collection and Ethical Statements

In French Guiana, non‐human vertebrate samples were collected and used in accordance with an international CITES permit (Convention on International Trade in Endangered Species of Wild Fauna and Flora; permit FR973A) according to French legislation. Following the sharing policy in French Guiana, non‐human mammal samples are registered in the collection JAGUARS (https://kwata.net/gestion‐collection‐biologique/, CITES reference: FR973A) supported by the Kwata NGO (accredited by the French Ministry of the Environment and the Prefecture of French Guiana, Agreement R03‐2019‐06‐19‐13), Institut Pasteur de la Guyane, DGTM Guyane, Collectivité Territoriale de la Guyane, and validated by the French Guianan prefectoral decree n°2012/110.

In Costa Rica, the animal study was reviewed and approved by the Institutional Committee for the Care and Use of Animals (Comité Institucional para el Cuidado y Uso de los Animales) of the Universidad de Costa Rica, and adhered to the Costa Rica legal requirements (collection permit number: MINAET‐SINAC, Costa Rica: 042‐2012‐SINAC).

In Brazil, the collected animals were dead and part of the epidemiology study of the Rio Grande do Sul State Health Secretariat, with a license issued by the Ministry of Health.

In Colombia, ethical approvals for the collection of fecal and blood samples as well as the collection of vectors were obtained by the Universidad de los Andes, the National Environmental Licensing Authority of Colombia (ANLA) and the Centers for Disease Control and Prevention (permits numbers: 2017025578‐1‐000, 2017043863‐1‐000, 2017065795‐1‐000, 2017013727‐1‐000, 2017052943‐1‐000, 2017081458‐1‐000, 2017108650‐1‐000).

In Argentina, permits were obtained from National Parks to conduct this research (IF 2022 85832064 APN DRNEA # APNAC PROJECT NUMBER 541 and IF 2022 85832064 APN DRNEA # APNAC PROYECT NUMBER 544).

### 4.2 DNA Extraction and *Plasmodium* Screening

For each of the 719 samples collected from NHPs in Latin America, genomic DNA was extracted using the Qiagen DNeasy Blood and Tissue Kit according to the manufacturer's recommendations. *P. simium* and *P. vivax* samples were identified by amplification of *Plasmodium cytochrome b* using nested PCR, as described in Prugnolle et al. (2010). The reaction products were visualized on 1.5% agarose gels stained with EZ‐vision.

For *P. simium* or *P. vivax* positive samples, selective whole‐genome amplification (sWGA) was performed to enrich submicroscopic levels of *Plasmodium* DNA, following the protocol described by Cowell et al. (2017). This technique preferentially amplifies *P. vivax* and *P. simium* genomes from a set of target DNAs and reduces host DNA contamination. For each sample, DNA amplification was carried out using the strand‐displacing phi29 DNA polymerase and *P. vivax* ‐specific primers that target short (6 to 12 nucleotides) motifs commonly found in the *P. vivax* genome (PvSet1 (Cowell et al. 2017; Sundararaman et al. 2013)). Although *P. vivax* ‐specific, these primers have already been used successfully to amplify other *Plasmodium* species phylogenetically close to *P. vivax* such as *P. vivax‐like* identified in African great apes (Daron et al. 2021). About 30 ng of input DNA was added to a 50 μL reaction mixture containing 3.5 μM of each sWGA primer, 30 U of phi29 DNA polymerase enzyme (New England Biolabs), 1× phi29 buffer (New England Biolabs), 4 mM deoxynucleotide triphosphates (Invitrogen), 1% bovine serum albumin, and sterile water. DNA was amplified in a thermal cycler with the following program: a ramp down from 35°C to 30°C (10 min per degree), 16 h at 30°C, 10 min at 65°C, and hold at 4°C. For each sample, the products of the two amplifications (one per primer set) were purified with AMPure XP beads (Beckman Coulter) at a 1:1 ratio according to the manufacturer's recommendations and pooled at equimolar concentrations. Samples with the highest concentration of parasite genome were selected after qPCR analysis using a Roche LightCycler 96 with the following program: 95°C for 10 min; 40 cycles of 95°C for 15 s, 60°C for 20 s, and 72°C for 20 s; 95°C for 10 s; and 55°C for 1 min. Each sWGA library was prepared using the two pooled amplification products and the Nextera XT DNA kit (Illumina), following the manufacturer's protocol. Then, samples were pooled, clustered, and sequenced on one lane of a Illumina Novaseq‐6000 S4 with 2 × 150‐bp paired‐end reads.

### 4.3 Short‐Read Mapping, SNP Calling, and Data Compilation

Three newly sequenced *P. vivax* isolates collected in Colombian monkeys were added to a compilation of previously published genomic datasets: (i) 621 *P. vivax* samples from Daron et al. (2021), Benavente et al. (2021), the MalariaGEN *P. vivax* Genome Variation project (Adam et al. 2022), Mourier et al. (2021), and Lefebvre et al. (2025); (ii) 31 *P. simium* samples from Mourier et al. (2021), de Oliveira, Rodrigues, Duarte, et al. (2021), and Ibrahim et al. (2023); and (iii) two *P. vivax‐like* genomes collected on Nigeria‐Cameroon chimpanzees ( *Pan troglodytes ellioti* ), from Loy et al. (2018) (Figure S1). Short read archives (SRA) from the literature were retrieved from NCBI (accession numbers in Table S1).

Short reads were trimmed to remove potential lingering adapters and preprocessed to eliminate low‐quality reads (*−quality‐cutoff = 30*) using the *cutadapt* program (Martin 2011). Reads shorter than 50 bp and containing “N” were discarded (*−minimumlength = 50 –max‐n = 0*). Cleaned paired‐end reads were mapped to the *P. vivax* reference genome PVP01 (Auburn et al. 2016) using *bwa‐mem* (Li and Durbin 2009). Duplicate reads were marked using the *MarkDuplicates* tool from the *Picard tools* v2.5.0 (broadinstitute.github.io/picard/) with default options. Local realignment around indels was performed using the *IndelRealigner* tool from *Genome Analysis Toolkit* (*GATK* (McKenna et al. 2010), v3.8.0). Variants were called using the *HaplotypeCaller* module in *GATK* with the parameter *‐stand_call_conf* equals to a Phred‐scaled confidence score of 10. Lastly, the different isolated variant call format (VCF) files were merged using the *GATK* module *CombineGVCFs*.

### 4.4 Data Filtering

For all samples ( *P. vivax* and *P. simium*) from Mourier et al. (2021), de Oliveira, Rodrigues, Duarte, et al. (2021), and Ibrahim et al. (2023), whether *P. vivax* or *P. simium*, the same filters used in Lefebvre et al. (2025) were applied. Thus, samples with > 50% missing data were removed. As several strains can infect the same host, the within‐host infection complexity was assessed with the *F*_*WS*_ metric (Amegashie et al. 2020), calculated using *vcfdo* (github.com/IDEELResearch/vcfdo; last accessed July 2022). Samples with mono‐clonal infections (i.e., *F*_*WS*_ > 0.95) were kept (Table S4). Highly related samples and clones could generate spurious signals of population structure, biased estimators of population genetic variation, and violated the assumptions of the model‐based population genetic approaches used in this study (Wang 2018). Therefore, the relatedness between haploid genotype pairs was measured by estimating the pairwise fraction of the genome in IBD using the *hmmIBD* program (Schaffner et al. 2018), with the default parameters for recombination and genotyping error rates, and using the allele frequencies estimated by the program. Within each country and species, isolate pairs that shared > 50% of IBD were considered highly related. Only the strain with the lowest amount of missing data was retained in each family of related samples.

The final dataset included 429 samples: 404 *P. vivax* isolates, 19 *P. simium* isolates, 4 *P. vivax* isolates from NHPs, and 2 *P. vivax‐like* isolates used as outgroup species in phylogenetic analyses.

For the VCF with 426 samples (without the three newly sequenced samples), only bi‐allelic SNPs with less than 50% missing data and a genotype quality (GQ) score above 30 were kept. SNPs with a minimum average depth coverage of 5× and a maximum average depth of 152× on all samples were kept. Singletons were removed because they can be sequencing errors. This dataset based on hard filters (*dataset_HF*) included 819,652 SNPs with a mean density of 38.29 SNPs per kilobase (Figure S1).

For the *ANGSD* software suite that uses the genotype likelihood (GL or GQ) rather than the actual genotype calling (Korneliussen et al. 2014), all sites with a genotype quality > 20, a maximum depth of 155× per sample, a minimum total depth of 5× on all samples, and present in at least 5 individuals were kept. Only bi‐allelic SNPs with a *p*‐value of 1e‐6 were kept. A total of 20,844,131 sites from the core genome were considered, including 1,286,079 variants (65.22 SNPs/kb). This approach allowed considering low to very low coverage samples (Korneliussen et al. 2014; Lou et al. 2021) and led to a dataset that included all 429 samples (*dataset _GL*). All filtration steps for both datasets are detailed in Figure S1.

### 4.5 Population Structure and Relationships Among Populations

The principal component analysis (PCA) and the individual genetic ancestry analyses were carried out with *ANGSD* v.040 and *PCAngsd* v0.98, after selecting only biallelic SNPs present in the *P. vivax* core genome and excluding SNPs with a minor allele frequency (MAF) < 5% from *dataset_GL*. Variants were LD‐pruned to obtain a set of physically unlinked variants using *ngsld* v1.1.1 (Fox et al. 2019), with a threshold of *r* ^2^ = 0.5 and windows of 0.5 kb. In total, these analyses included 247,890 SNPs for 425 individuals (the *P. vivax‐like* outgroup was not considered). For the ancestry analyses, using *PCAngsd*, a number of clusters (*K*) ranging from 2 to 8 were tested. The best K value was inferred based on the broken‐stick eigenvalues plus one (Figure S3), following the recommendations of Meisner and Albrechtsen (2018). The results were post‐processed using *pong* v1.5 (Behr et al. 2016) to compute the ancestry proportions.

The maximum likelihood (ML) phylogenetic tree was obtained with *IQ‐TREE* (Nguyen et al. 2015) using the best‐fitted model determined by *ModelFinder* (Kalyaanamoorthy et al. 2017). Considering the *dataset_HF*, only SNPs with ≤ 10% missing data and samples with < 50% missing data were retained, resulting in 48,070 SNPs in the core genome for 404 samples. As the dataset was composed only of SNPs and no invariant sites, the ascertainment bias correction was added to the tested models, following the *IQ‐TREE* user guide recommendations. According to the Akaike information criterion (Akaike 1974), the best model of nucleotide evolution was the general time reversible (GTR) model that integrates unequal rates and unequal base frequency (Tavaré 1986). Node support was estimated using the Ultrafast Bootstrap Approximation (UFboot) (Minh et al. 2013) and SH‐aLRT methods (Guindon et al. 2010). Nodes were considered as highly supported only if SH‐aLRT ≥ 80% and UFboot ≥ 95%, following the thresholds by Guindon et al. (2010) and Minh et al. (2013).

PCA and genetic ancestry analyses were performed focusing only on the *P. simium* samples to investigate the genetic substructure within this group. As these analyses require a data set with unlinked variants, SNPs from the *dataset_GL* (with only *P. simium* samples) were LD‐pruned with *ngsld* v1.1.1 (Fox et al. 2019), with a threshold of *r* ^2^ = 0.5 and windows of 0.5 kb. In total, these analyses included 44,911 SNPs for 19 individuals. The PCA was carried out with *ANGSD* v.040 and *PCAngsd* v0.98, after selecting only biallelic SNPs present in the *P. vivax* core genome and excluding singletons. Then, individual genetic ancestry was estimated using the *PCAngsd*. Different cluster (*K*) numbers were tested from 1 to 5. The optimal *K* value was estimated based on the broken‐stick eigenvalues plus one (Figure S3), following the recommendations of Meisner and Albrechtsen (2018), and the plots were generated with *pong* (Behr et al. 2016).

For the pairwise‐IBD network, the complete *dataset_HF*, composed of 819,652 SNPs and 426 samples, was used. Pairwise‐IBD values among pairs of isolates were estimated using the *hmmIBD* program (Schaffner 2019), with the default parameters for recombination and genotyping error rates, and using the allele frequencies estimated by the program. The pairwise‐IBD relationships among isolates were visualized as a network that displayed only pairwise IBD relationships with values ≥ 5%. The network was plotted using the *igraph* package in R (Csardi and Nepusz 2006) and default options.

Population graphs were built using *TreeMix* (Pickrell and Pritchard 2012) and *AdmixtureBayes* (Nielsen et al. 2023). First, the *dataset_HF* was pruned using PLINK (Chang et al. 2015) with a threshold of *r* ^2^ = 0.5, a sliding window of 50 SNPs and a step of 10 SNPs (396,835 SNPs remaining). All SNPs with missing data were removed. The final dataset used in these analyses included 11,907 SNPs for 18 ingroup populations and one outgroup population (*P. vivax‐like*).

The number of migration events (*m*) in *TreeMix* that best fitted the data was calculated by running the analysis 15 times for each *m* value, with *m* ranging from 1 to 10. The optimal *m* value was estimated using the *OptM* R package (Fitak 2021) and the Evanno method (Figure S6). Then, a consensus tree with bootstrap node support was obtained by running 100 replicates of *TreeMix* and postprocessing them using the *BITE* R‐package (Milanesi et al. 2017) for the optimal *m* values, following Daron et al. (2021) and Lefebvre et al. (2023).

For the *AdmixtureBayes* analysis, three independent runs were performed, each one including 40 coupled Monte Carlo Markov Chains (MCMC) and 250,000 steps. The results of these chains were analyzed after a burn‐in of 50% and a thinning step of 10, using the default parameters. Convergence across the trees was assessed using the Gelman‐Rubin (GR) statistics for the model parameters (i.e., posterior probability, branch lengths and number of admixtures), as recommended by Nielsen et al. (2023). Runs were deemed convergent if the GR statistics for the model parameters converged toward the value of 1 along the MCMC steps (Figure S5). The retained network was common among the three best networks in each chain that converged, based on their posterior probabilities.

The admixture *f*_*4*_‐statistics were computed with *ADMIXTOOLS 2* v2.0.0 (Maier and Patterson 2024) and the same dataset used for *TreeMix* and *AdmixtureBayes*.

### 4.6 Assessment of Low‐Coverage Bias on Population Structure Inference

To assess the effect of low sequencing coverage on PCA and PCA‐based admixture analyses, we downsampled four high‐coverage *P. vivax* genomes (*mauritania*, *p1542.PV.Ethiopia*, *p1616.PV.Thailand*, and *Brazil_Belem*) to an average depth of 1.32× (Table S5) using *SAMtools* v1.21 (Danecek et al. 2021). To capture stochastic variability introduced by downsampling, we generated 100 replicates per sample. Each replicate was incorporated into a PCA using the same LD‐pruned SNP set employed in the global PCA of all *P. vivax* and *P. simium* samples (Figure 2), adding one replicate per genome at a time. For each replicate, Euclidean distances were calculated in *R* v4.3.1 (R Core Team 2023) to quantify divergence from the original high‐coverage placement (Figure S2).

### 4.7 Demographic History of American *P. simium*

The nucleotide diversity (*π*) values were estimated using *pixy* v1.2.7 (Korunes and Samuk 2021), in a non‐overlapping sliding window of 500 bp along the genome based on the *dataset_GL*. The sample size was standardized (i.e., 10 randomly chosen isolates for each population) to obtain values that could be compared.

*Relate* (Speidel et al. 2019) was used to infer historical changes in *N*_*e*_ and to estimate the divergence time between populations. The ancestral/derived states of 1,267,897 sites from the *dataset_GL* were defined using the consensus sequence of the outgroup (*P. vivax‐like*). Only the core genome regions as defined in Daron et al. (2021) were considered; the rest was masked. As the *P. simium* samples were not enough (*n* = 19) to generate a *P. simium*‐specific genetic map, the *P. vivax* genetic map from Lefebvre et al. (2025) was used with a mutation rate of 6.43 × 10^−9^ mutations/site/generation and a generation time of 5.5 generations/years (Daron et al. 2021). Coalescence rates were calculated from 10 to 1,000,000 generations ago (options *‐‐bins 0,7,0.25*).

Then, MSMC‐IM (Wang et al. 2020), based on the coalescent rates estimated within and between two populations from *Relate* (Speidel et al. 2019), was used to fit an isolation‐with‐migration (IM) model. The scripts to convert *Relate* results to *MSMC‐IM* were kindly provided by E. Patin and D. Liu from the Human Evolutionary Genetics team at the Institut Pasteur, Paris, France. The migration rates were inferred between *P. simium* clusters and two *P. vivax* populations (Brazil and Mexico). These *P. vivax* populations were selected based on their known evolutionary history and our population structure results. Brazilian and Mexican *P. vivax* were included due to their geographic proximity to *P. simium* and genetic similarity to *P. simium*, respectively. The mutation rate was the one used for *Relate*, and the population sizes were derived from the *Relate* results.

### 4.8 Positive Selection Detection

Given the low sample size in the two *P. simium* genetic clusters (*n* = 13 and *n* = 6), they were combined for the selection scan analyses with *rehh* (Gautier et al. 2017) and *Relate* (Speidel et al. 2019), following Hupalo et al. (2016) and Lefebvre et al. (2023).

The haplotype‐based tests (*iHS, XP‐EHH*, and *Rsb*) were used to detect evidence of recent selective sweeps, using *rehh* (Gautier et al. 2017) and *dataset_HF*. The significance threshold was set at −log (*p‐value*) = 4, as recommended by Gautier et al. (2017). For *XP‐EHH* and *Rsb*, the *P. simium* samples were compared with Brazilian *P. vivax* samples and only SNPs with negative standardized values (i.e., indicating positive selection for the *P. simium* cluster rather than for *P. vivax* ) were considered. The ancestral/derived states of 378,441 SNPs from *dataset_HF* were defined using the consensus sequence of the two outgroup *P. vivax‐like* isolates. The Brazilian population was chosen due to its larger sample size (*n* = 29). The Mexican population did not have a large enough sample size (*n* = 9) to be used as a reference population to detect selection signals (Pickrell et al. 2009).

The *Relate* program used to estimate the coalescent rates in the demographic section was also used to detect positive selection along the genome by identifying genomic regions with significantly higher coalescent rate values than the rest of the genome. The significance threshold was set at –log (*p‐value*) = 4, to take into account the multiple tests.

The population branch‐site (*PBS*) values were calculated for each *P. simium* cluster, with *ANGSD* v0.40 in sliding windows of the genome (windows of 1 kb with a sliding step of 500 pb), based on *dataset_GL*. The outgroup populations were *P. vivax* from humans in Brazil and Colombia. All windows with < 20 SNPs were removed to avoid extreme values caused by a low SNP number. The 0.1% most extreme values were considered evidence that the genomic regions displayed signs of selection in our *P. simium* clusters.

Once selection signals were detected, the identified genes were annotated using the general feature format (GFF) file (*PlasmoDB‐50_PvivaxP01.gff*, available on *PlasmoDB*) and the intersect function of *BEDtools* v2.26.0 (Quinlan and Hall 2010). Additional information (e.g., gene name, function, and biological process) was retrieved from *PlasmoDB* (plasmodb.org, accessed in April 2024).

## Funding

Project ORA in the LabEx CEBA, ANR‐10‐LABX‐25‐01 (V.R.). JCJC GENAD, ANR‐20‐CE35‐0003 (V.R.). MICETRAL, ANR‐19‐CE350010 (F.P.). The United States (U.S.) Agency for International Development and the U.S. Centers for Disease Control and Prevention (CDC) for the project entitled “Evaluation of ZIKV potential to establish a sylvatic transmission cycle in Colombia” (C.G.R., A.L., S.R.).

## Conflicts of Interest

The authors declare no conflicts of interest.

## Supporting information


**Figures S1–S8:** eva70222‐sup‐0001‐FigureS1‐S8.pdf.

**Tables S1–S5:** eva70222‐sup‐0002‐TableS1‐S5.xlsx.

## Data Availability

The sequences of the newly sequenced and analyzed simian *P. vivax* samples have been deposited on NCBI (Bioproject PRJNA116416). SNP data files (VCF format) produced in this study as well as the related metadata and documentation are available in the DataSuds repository (IRD, France) at https://doi.org/10.23708/RCOGR6. The scripts used in this study are available in this github repository: https://github.com/MargauxLefebvre/EvoHistory_Simium.
